# A fast X-ray shutter for high-power beams

**DOI:** 10.1107/S1600577526000482

**Published:** 2026-02-18

**Authors:** Thierry Lachat, Benedikt Rösner, Ana Diaz, Xavier Donath, Andreas Menzel, Mirko Holler

**Affiliations:** a Paul Scherrer Institute, PSI Center for Photon Science, Forschungsstrasse 111, 5232Villigen PSI, Switzerland; Brazilian Synchrotron Light Laboratory, Brazil

**Keywords:** fast X-ray shutters, piezo shutters, beamline instrumentation

## Abstract

We present the development of a piezo-actuated shutter system designed for high-power broadband X-ray beams at fourth-generation synchrotron sources. The device combines a flexure-based mechanical design with efficient water cooling, achieving full open–close transitions in 2 ms while reliably withstanding continuous thermal loads exceeding 20 W. This performance enables safe operation with multilayer monochromators, which introduce higher heat loads than conventional optics such as Si(111) monochromators. The shutter provides an open aperture of 1.9 mm, is fully ultrahigh vacuum compatible, and was validated through mechanical and thermal characterization under realistic operating conditions. Its millisecond-scale actuation allows precise blocking of the beam during idle phases such as sample alignment or repositioning, thereby minimizing radiation damage and maximizing the effective use of the delivered photon flux. This makes the system particularly well suited for high-throughput scanning and imaging techniques, including ptychography, tomography, scanning small-angle X-ray scattering and protein crystallography, on modern synchrotron beamlines.

## Abstract 2

A fast thermally resilient X-ray shutter achieves 2 ms switching under heat loads above 20 W, enabling efficient photon use and minimizing radiation damage at fourth-generation synchrotron sources.

## 1. Introduction

X-ray-based characterization methods are fundamental in both materials and life sciences. Today’s cutting-edge X-ray facilities are fourth-generation synchrotron light sources built on the multibend achromat (MBA) concept, which offers vastly improved brilliance and coherence compared with earlier generations (Eriksson *et al.*, 2008 ▸). However, increased brilliance also increases the risk of radiation damage to sensitive samples. In general, a sample must not be irradiated while data are not being collected. The speed of the shutter is particularly important in synchrotron techniques where the sample is illuminated but not actively measured, such as during sample alignment, repositioning, fly scan turnarounds or detector readout phases. In such cases, unnecessary irradiation must be minimized to reduce radiation damage while maintaining experimental efficiency. Unnecessary exposure can be prevented by using a fast mechanical shutter that blocks the beam when no data are being acquired (Howells *et al.*, 2009 ▸; Garman, 2010 ▸; Nave & Garman, 2005 ▸; LeGrand *et al.*, 1989 ▸; Kujala *et al.*, 2014 ▸).

Several commercial solutions exist, offering a range of actuation principles and response times. The Colibri X-ray fast shutter by Arinax (https://www.arinax.com/colibri-x-ray-fast-shutter/) achieves millisecond-scale switching, making it suitable for time-resolved applications where high-speed beam blocking is essential. Similarly, the XRS25H shutter from Uniblitz (https://www.uniblitz.com/products/xrs25h/) is based on a photographic-style mechanism and delivers opening times around 20 ms with a 25 mm aperture, suitable for lower-flux or monochromatic beamlines. The commercial shutters mentioned above are devoted to protein crystallography applications. Piezoelectric shutters are particularly attractive for integration into beamlines requiring fast modulation without the thermal inertia or acoustic noise typical of voice-coil or motor-driven devices. Piezo shutters offer distinct advantages in terms of speed, compactness and precision. Commercial shutters are, for example, available from Cedrat Technolgies (https://cedrat-technologies.com/) and Dynamic Structures and Materials (DSM) (https://www.dynamic-structures.com/). Both companies offer a range of fast actuators and shutters tailored for high-speed X-ray applications. The flexure-based mechanisms are designed for robust vacuum-compatible performance, with response times in the low millisecond range depending on the exact configuration. Finally, for modular integration into beamline setups, Huber Diffraktionstechnik (https://www.xhuber.com/de/produkte/4-zubehoer/43-strahl/beam-conditioning-unit/shuttermodule/) provides shutter modules as part of their beam conditioning units. These are designed for compatibility with high-precision stages and optics.

The devices mentioned thus far are designed for low-power monochromatic beams. With the growing use of multilayer monochromators – which offer bandwidths around 10^−2^ compared with the narrow 10^−4^ of traditional Si(111) monochromators – thermal loads are higher, and thus shutter requirements become more demanding (Attwood & Sakdin­awat, 2017 ▸; André *et al.*, 2002 ▸; Chu *et al.*, 2002 ▸; ESRF, 2003 ▸; Koyama *et al.*, 2022 ▸; Singhapong *et al.*, 2024 ▸). Innovative mechanical shutter designs have been proposed which might meet these requirements. For instance, LeGrand and co-workers described a rotating disc chopper capable of ultrafast periodic operation on the picosecond timescale (LeGrand *et al.*, 1989 ▸). Similarly, Kujala and co-workers presented a voice-coil actuated white-beam shutter that achieves 32 ms switching time and is specifically designed for topography at the Advanced Photon Source (Kujala *et al.*, 2014 ▸).

In the present work, we describe the development of a high-speed shutter system tailored for high-flux polychromatic X-ray beams. It addresses both the speed and thermal resilience needed for modern beamlines that employ advanced scanning techniques and broadband optics. The shutter will be implemented on the upgraded coherent small-angle X-ray scattering (cSAXS) beamline at the Swiss Light Source (SLS) at the Paul Scherrer Institute, Switzerland.

## 2. The cSAXS beamline at SLS2.0

Since 2008, the coherent small-angle X-ray scattering beamline (cSAXS) has supported experiments in SAXS, scanning SAXS (Bunk *et al.*, 2009 ▸) and X-ray ptychography, in particular ptychographic X-ray tomography (Holler *et al.*, 2014 ▸), across a wide range of scientific fields. With the upgrade of the SLS (Streun *et al.*, 2018 ▸) the cSAXS beamline undergoes an upgrade as well (Roesner *et al.*, 2024 ▸). In future, it will offer two options for monochromatizing the beam: a Si(111) channel-cut monochromator and a multilayer monochromator. With the broadband multilayer option, a maximum power of 5 W is expected after the monochromator. Previously, a commercial in-vacuum fast shutter was integrated, the XRS1-800 model from DSM. This piezoelectrically driven shutter offers an open aperture of 0.7 mm and an open–close time of 3.4 ms. It was in continuous use for approximately seven years at typical rates in the 10 Hz range without any failures. It will remain available, but can only be employed with the narrowband beam because it is not suitable for high X-ray powers. Thermal expansion at higher power levels leads to structural deformation of the flexure structures, ultimately causing the shutter to remain permanently closed. Cooling structures are required at the expected power levels, which on the one hand must not significantly increase the mobile mass in order to maintain short actuation times. On the other hand, very effective cooling is required for the flexure structures not to deform and the open aperture to decrease.

Attempts to design a lightweight cooling structure with sufficient thermal performance proved unsatisfactory. Therefore, we have developed a new shutter mechanism based on a structural design tolerant of temperature fluctuations. All design parameters exceed the current needs of the upgraded cSAXS beamline, with the goal of making the shutter attractive to other beamlines as well. The key design specifications are as follows:

(i) Symmetric actuation to minimize excitation of surrounding mechanical components.

(ii) Continuous heat load of up to 22 W (cSAXS requirement: 5 W).

(iii) Resonance frequency in the 1 kHz range, targeting 1 ms actuation time.

(iv) Open aperture: > 2 mm (cSAXS requirement: 1 mm).

(v) Ultrahigh vacuum compatible.

(vi) Dimensions of the module: width 150 mm × height 50 mm × depth 25 mm.

## 3. Mechanical design

**Figure 1:**
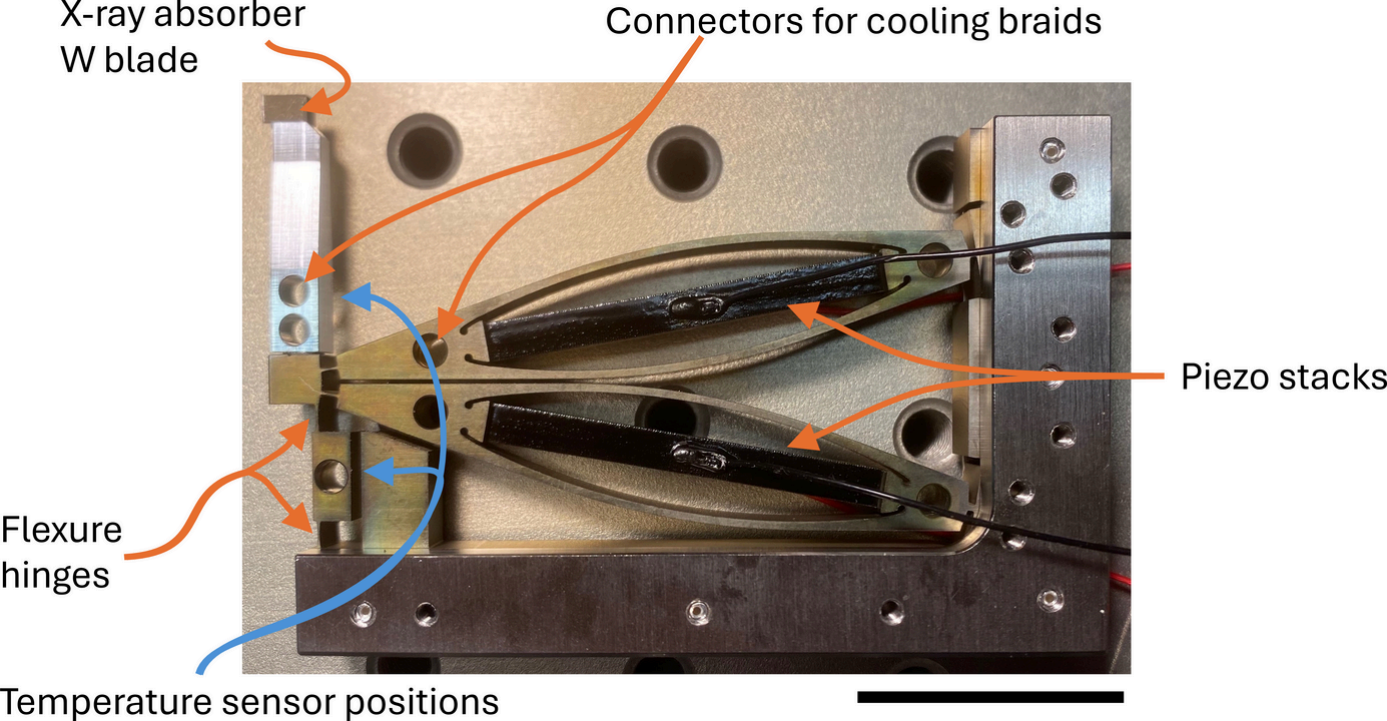
A half shutter module. The X-rays will be absorbed at the tip of the lever structure on the left. The lever is connected via two flexure hinges to the piezo actuators, which operate in inverse directions. An expansion of the upper piezo and a contraction of the lower piezo will result in a movement of the lever to the left, which corresponds to a closing motion. Two such modules are combined for a full shutter. Scale bar: 25 mm.

The design is based on a flexure structure manufactured from titanium that is operated by two piezoelectric actuators. Fig. 1 ▸ shows a photograph of one half of the shutter module. The X-rays will be absorbed at the tip of the lever structure on the left. The lever is connected via two flexure hinges to the piezo actuators, which operate in inverse directions. An expansion of the upper piezo and a contraction of the lower piezo will result in a movement of the lever to the left, which corresponds to a closing motion. A video of a moving half shutter is available as supplementary video 1. Two such modules are combined to form a complete unit.

**Figure 2:**
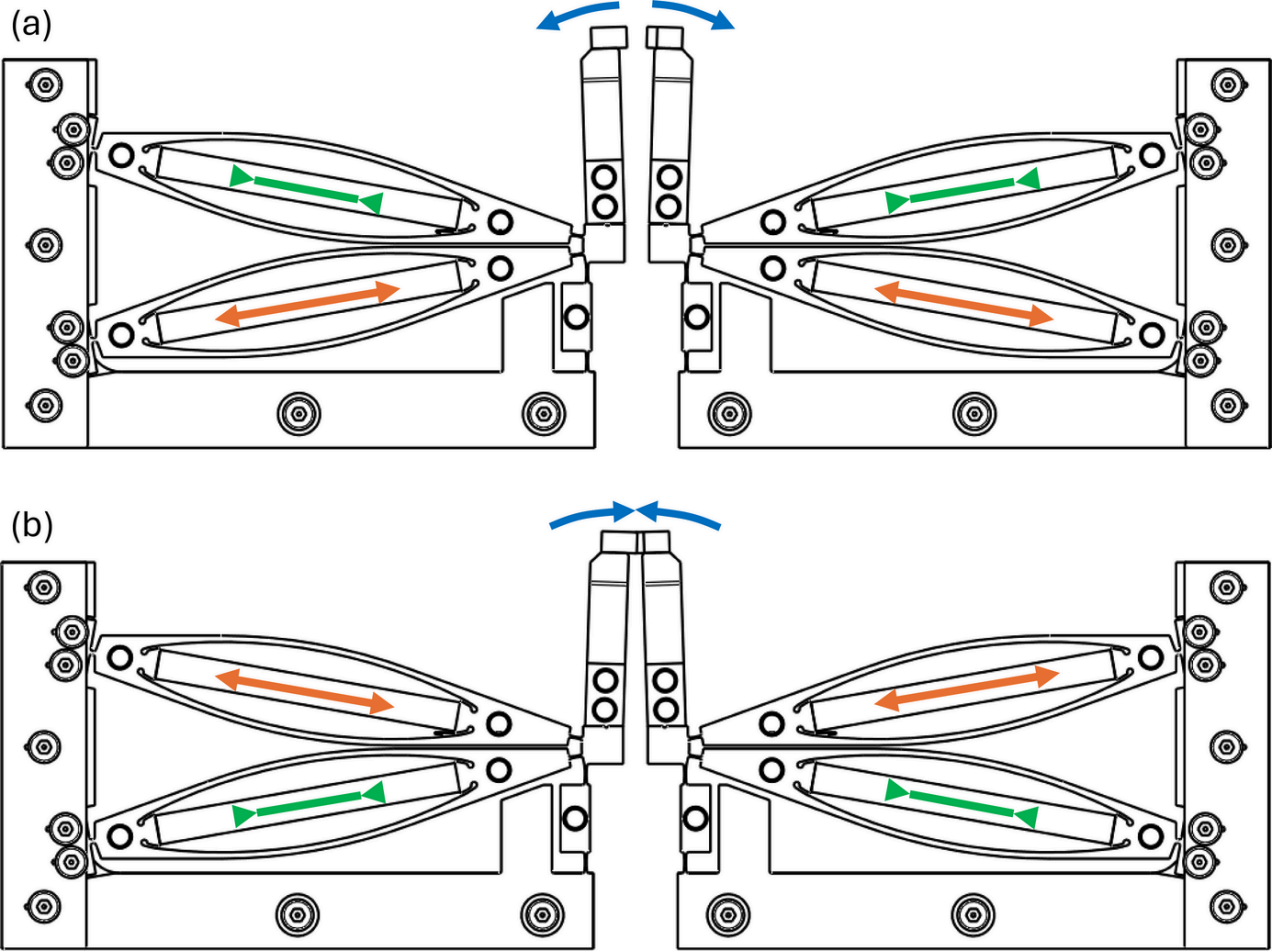
Schematic diagram of the shutter movements. The shutter shown in (*a*) is in the open state, with the lower piezo elements expanded (indicated by the orange arrows) and upper piezo elements contracted (indicated by the green arrows). The shutter shown in (*b*) is in the closed state.

**Figure 3:**
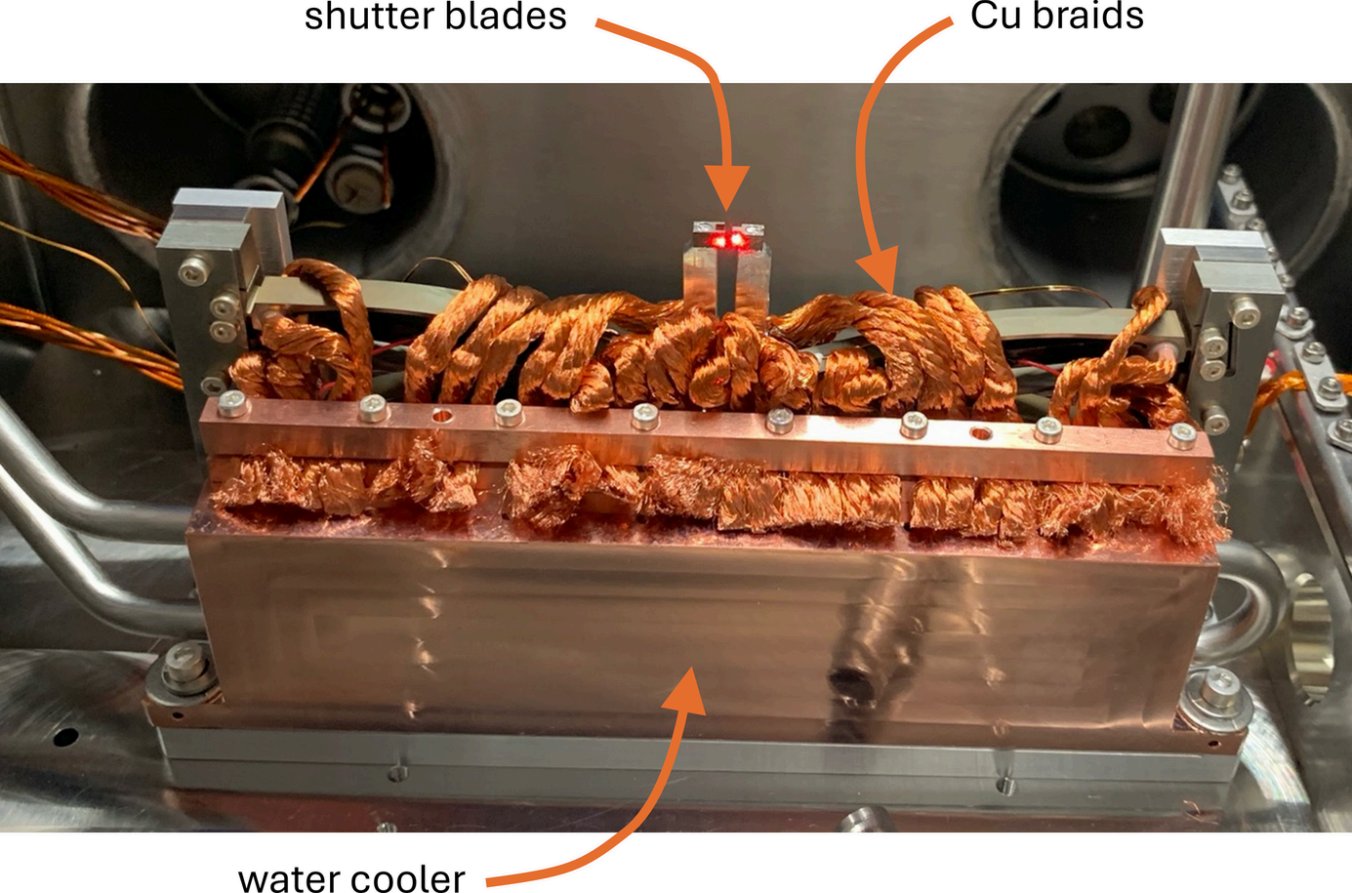
Photograph of the fully assembled shutter. The water-cooled copper block is visible in front, as well as all the cooling braids connected to the shutter mechanism. The copper braids are glued though the circular holes of the shutter module and mechanically clamped to the cooled copper block. The laser spot visible on the shutter blades was used to characterize the shutter and measure the actuation speed. Total dimensions of the module: width 150 mm × height 50 mm × depth 25 mm. Supplementary video 2 shows the shutter in actuation.

Fig. 2 ▸ illustrates the mechanical working principle of the shutter unit. Copper braids connected to a water-cooled copper block will be implemented at all the circular holes indicated in Fig. 1 ▸. For good thermal conductivity and low mobile mass, the lever is made from aluminium. The flexure structure is wire-eroded from titanium, and the tip of the lever holds a tungsten blade with a thickness of 1 mm for good X-ray attenuation. The fully assembled shutter is shown in Fig. 3 ▸ and the geometry of the shutter blades is displayed in Fig. S1 of the supporting information. Absorbed power at the top of the lever will lead to a significant increase in its temperature, but the piezo driving structure is isolated by the flexure hinges from the hot shutter arm. Thermal expansion thus mainly occurs along the vertical direction, which is the uncritical direction and does not affect the aperture of the shutter mechanism.

Finite element analysis (FEM) shows the first resonance of 980 Hz in the direction of movement of the shutter arm. The second resonance is simulated at 1204 Hz. Two piezo stacks (Piezomechanik GmbH, PSt150/3.5·3.5/40) are mounted in each flexure structure, which also produces the required pre-tension to the piezo elements. The modelled full stroke of one half shutter arm is 1 mm. The measured range over the full voltage range of the piezo stacks (−30 V to 150 V) is 0.95 mm, which matches the expectation.

## 4. Mechanical characterization of the shutter

The shutter was driven by two linear piezo amplifiers (Model VF-500, DSM), each capable of delivering a peak current of 1000 mA. When excited with a sinusoidal signal, audible resonances were observed at 790 Hz and 1230 Hz. To characterize the shutter’s aperture and speed, a HeNe laser beam was used and the transmitted light intensity was recorded with a photodiode. The incoming beam was several millimetres in diameter and filtered by a rectangular aperture (1 mm × 3 mm in size) before the shutter.

Various electrical driving signals were tested using only half a shutter module, *i.e.* a single blade. Fig. S2 of the supporting information shows the mechanical response, *i.e.* blade position *versus* time, when the amplifier is driven with a simple step (edge) function. This broadband excitation leads to significant oscillations of the shutter blade, which are undesirable and take a long time to decay. The situation improves when a simple passive second-order low-pass filter is applied to the electrical drive signal, as shown in Fig. S3. With a cutoff frequency of 150 Hz, the filter achieves −30 dB attenuation at 850 Hz. This significantly suppresses the oscillations, although some residual ringing remains due to the sharp rising edge of the input signal. Nevertheless, the shutter might be suitable for some applications in this configuration.

To optimize the mechanical behaviour further, a custom driving signal was computed in Python at a 10 kHz sampling rate. It started from an edge function which was filtered by a 300 Hz low-pass filter and a notch filter at 790 Hz (*Q* factor of 50). The Python code is available in the supporting information, and Fig. S4 presents the synthesized drive waveform and the resulting shutter blade movement. The blade now follows the desired trajectory smoothly, without introducing oscillations.

**Figure 4:**
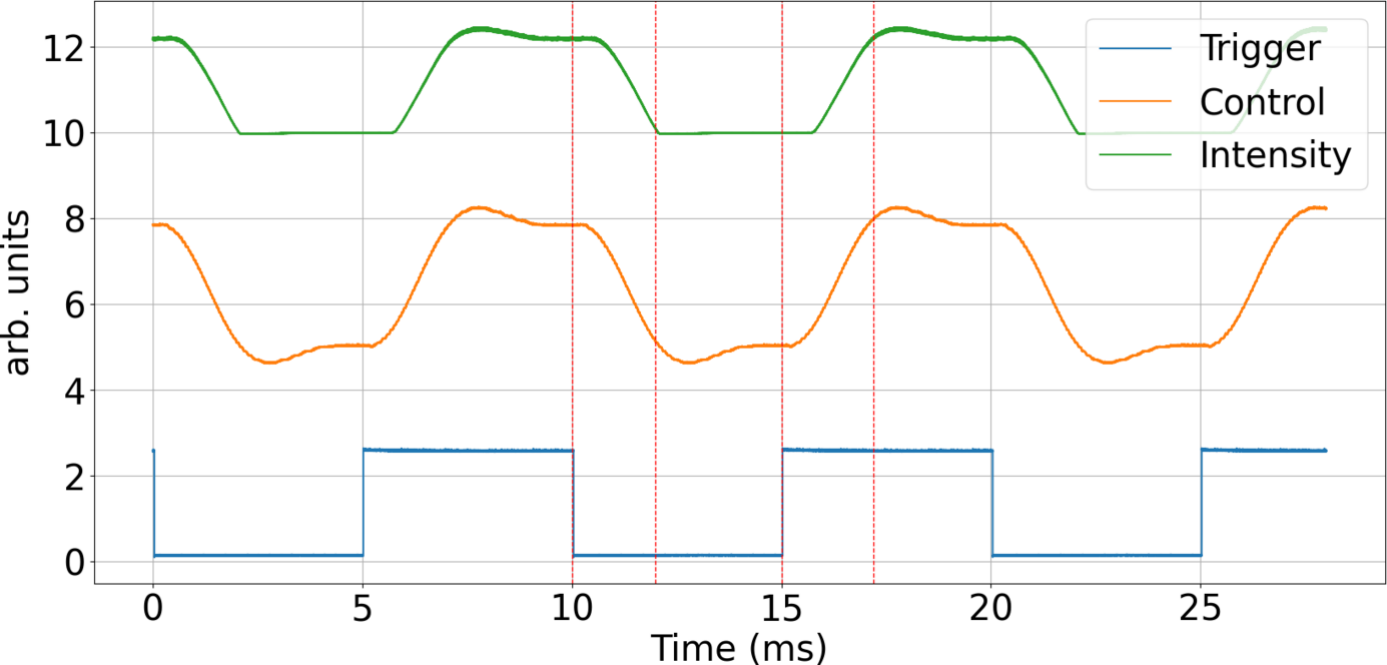
Operation of the shutter, showing the trigger signal (blue), the generated drive signal (orange) and the measured light intensity corresponding to the shutter opening and closing (green). The data reflect a fully assembled device with two opposing blades. The time for a full transition of the shutter is 2 ms. In the example shown, the shutter operates continuously at 100 Hz, but it can also be triggered asynchronously, *i.e.* opened and closed on demand.

For beamline operation, the full shutter (comprising two opposing blades) is controlled by an Arduino Due microcontroller. This device includes two on-chip digital-to-analogue converters (DACs) and is fast enough to generate the required drive signals. A digital input signal triggers the controller to output either the opening or closing waveform on DAC0 (upper piezo), with the corresponding inverted waveform sent to DAC1 (lower piezo). The amplifiers require an input voltage range of −1.5 V to 7.5 V. Therefore, a set of operational amplifiers is used to condition the DAC outputs by applying offset and gain corrections. A 1 kHz low-pass filter is added to suppress potential DAC noise. Fig. 4 ▸ shows the trigger input, the waveform generated at DAC0 and the measured light intensity at the photodiode.

The shutter’s maximum aperture as aligned in the present version is 1.5 mm, which is more than sufficient for the cSAXS beamline, where only 1 mm is required. The shutter’s closed position was aligned generously in this case, as evident in the sharp transitions observed in the intensity signal. The entire unit is mounted on a translation stage (SmarAct GmbH) and can be moved a few millimetres fully out of the beam. The braids are flexible while the water cooler is at a fixed position.

## 5. Thermal characterization of the shutter

**Figure 5:**
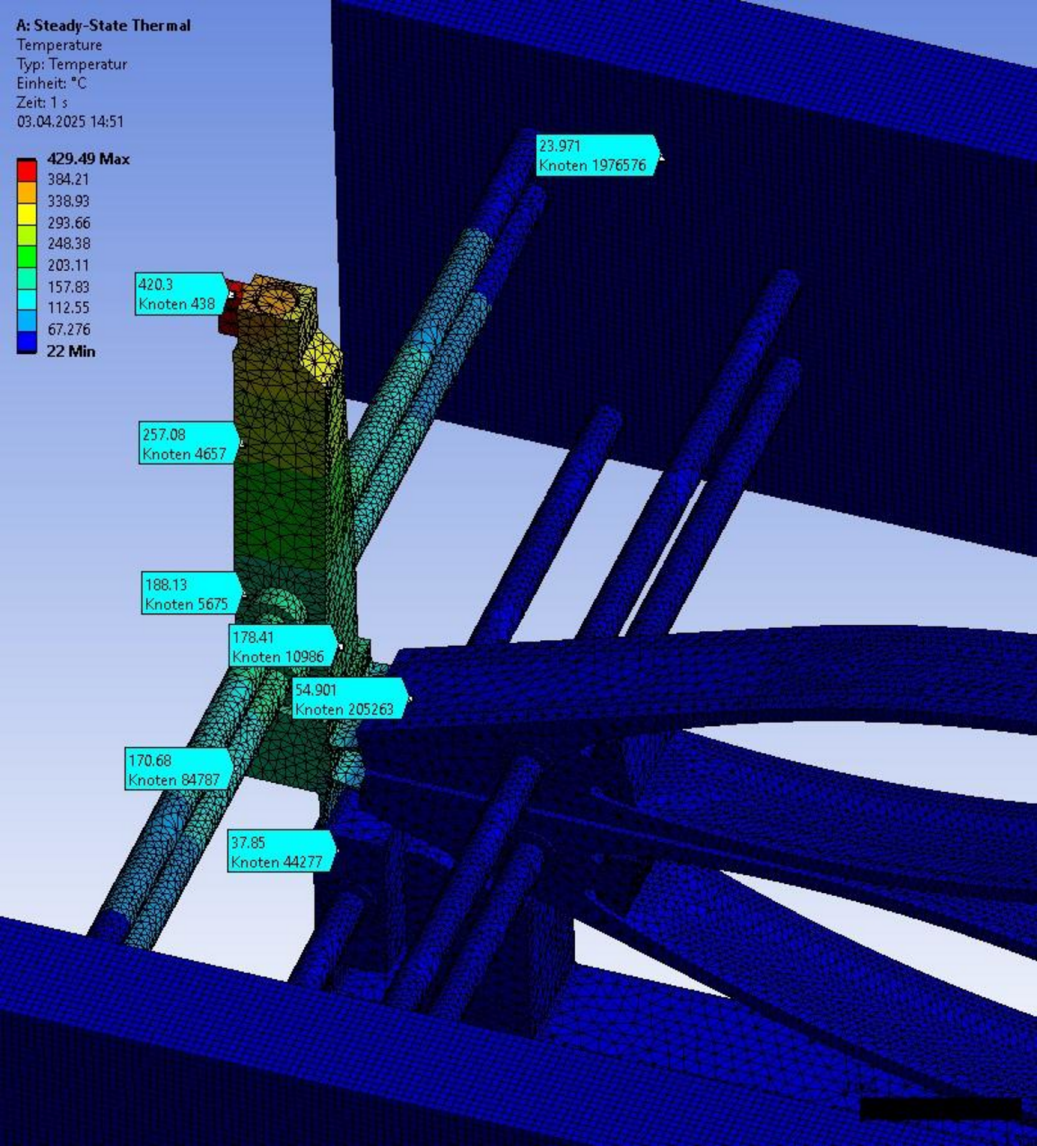
Thermal simulation of shutter temperatures with cooling water at 22°C and a thermal load of 22 W incident power to a single shutter blade. In typical applications, this heat is distributed across both blades. However, in the event of misalignment of either the assembly or the incoming beam, the full power could be incident on a single blade. Therefore, the simulation represents a worst-case scenario. The maximum expected temperature is 420°C, which remains well below the melting points of both tungsten (W) and aluminium (Al). Colour scale 22°C to 429°C. The turquoise labels indicate the simulated temperature at these particular positions.

The shutter is designed for a heat load of more than 20 W. To confirm this, steady-state FEM analysis has been performed using the software *Ansys*(https://www.ansys.com/). As boundary conditions, a cooling block temperature of 22°C was fixed and various incident power levels were applied to the tungsten absorber. In the case of misalignment of the X-ray beam, only one blade might be absorbing the full power. To simulate this worst case, one shutter blade was exposed to 22 W and the result of the simulation is shown in Fig. 5 ▸. Such power is expected on the TOMCAT beamline at SLS. The maximum temperature expected in this case is 429°C, which is well below the melting point of aluminium. The temperature of the base region reaches temperatures in the region of 150°C, but the flexure structure in contact with the piezo elements stays below 60°C, which is below the recommended maximum operating temperature of the piezo elements used.

On the cSAXS beamline, the expected heat load is below 10 W. Since the new beamline at SLS2.0 was not yet operational during the construction and testing phases of the shutter, an electrical heater was used to simulate the thermal load. The heater was attached to one of the aluminium levers of the shutter, delivering up to 12 W of power. Temperature measurements were conducted using PT100 sensors placed at two locations: directly on the lever near the heater, and on the structure at the base (see Fig. 1 ▸ for sensor positions). Table 1 ▸ summarizes the measured temperatures for various heating powers. All measurements were performed under vacuum conditions.

Temperatures were measured under steady-state conditions. The cooling water was initially switched off. At 8 W heating power, the base temperature increased to 43.1°C. With active cooling water at 20.5°C this temperature dropped to 26.6°C, clearly showing the effectiveness of the cooling mechanism. The power was then increased to 12 W. Fig. S5 shows the steady-state simulation at 12 W power. The maximum measured temperature of 163.2°C is higher than the simulated value of 142.8°C at 12 W. This discrepancy is probably due to suboptimal thermal contact between the copper braids and the water cooler. In the experimental setup, indium foil – a planned feature to improve the performance of the thermal interface in high-load versions – was not applied. As the expected power on the cSAXS beamline is below 10 W, indium foil will not be introduced in the current shutter version in the future.

## 6. Conclusion and outlook

We have developed a fast thermally resilient shutter system designed to meet the demanding requirements of high-brilliance broadband X-ray beamlines. The system combines piezoelectric actuation with a robust flexure-based mechanical design, achieving 2 ms transition times at an aperture of 1.9 mm and continuous operation under heat loads exceeding 20 W. Thermal and mechanical characterizations confirm both high-speed performance and heat-load compatibility under vacuum conditions.

Initially implemented on the upgraded cSAXS beamline at SLS2.0, the shutter is well suited to other beamlines. While the prototype is currently operational at our institute, we are developing the shutter into a commercial product in collaboration with Nanofaktur GmbH (https://www.nanofaktur.com/) to make it broadly accessible.

## Supplementary Material

Supplementary Information. DOI: 10.1107/S1600577526000482/tol5018sup1.pdf

Supplementary Movie 1. DOI: 10.1107/S1600577526000482/tol5018sup2.mov

Supplementary Movie 2. DOI: 10.1107/S1600577526000482/tol5018sup3.mov

## Figures and Tables

**Table 1:** Measured temperatures at various heating powers with and without water cooling

Heating power (W)	Water cooling	Temperature (°C)	Base temperature (°C)
1.5	Off	45.6	23.3
6	Off	106.6	36.1
8	Off	137.7	43.1
8	On	123.2	26.6
12	On	163.2	27.0

## Data Availability

Data are available within the article and supporting information.
