# Supplementary material for: A fast X-ray shutter for high-power beams
Source: J Synchrotron Radiat. 2026 Feb 18;33(Pt 2):314–8. doi: 10.1107/S1600577526000482 (PMC12947999; doi:10.1107/S1600577526000482)
Supplement: Supplementary file 1 [file s-33-00314-sup1.pdf]

JOURNAL OF SYNCHROTRON RADIATION

Volume 33 (2026)

Supporting information for article:

A fast X-ray shutter for high-power beams

Thierry Lachat, Benedikt Rösner, Ana Diaz, Xavier Donath, Andreas Menzel and Mirko Holler

A fast X-ray shutter for high power beams
Supplementary Information

Thierry Lachat [a], Benedikt Rösner [a], Ana Diaz [a], Xavier Donath [a], Andreas Menzel [a], and Mirko Holler [a]

[a]Paul Scherrer Institute, Forschungsstrasse 111, 5232 Villigen PSI, Switzerland

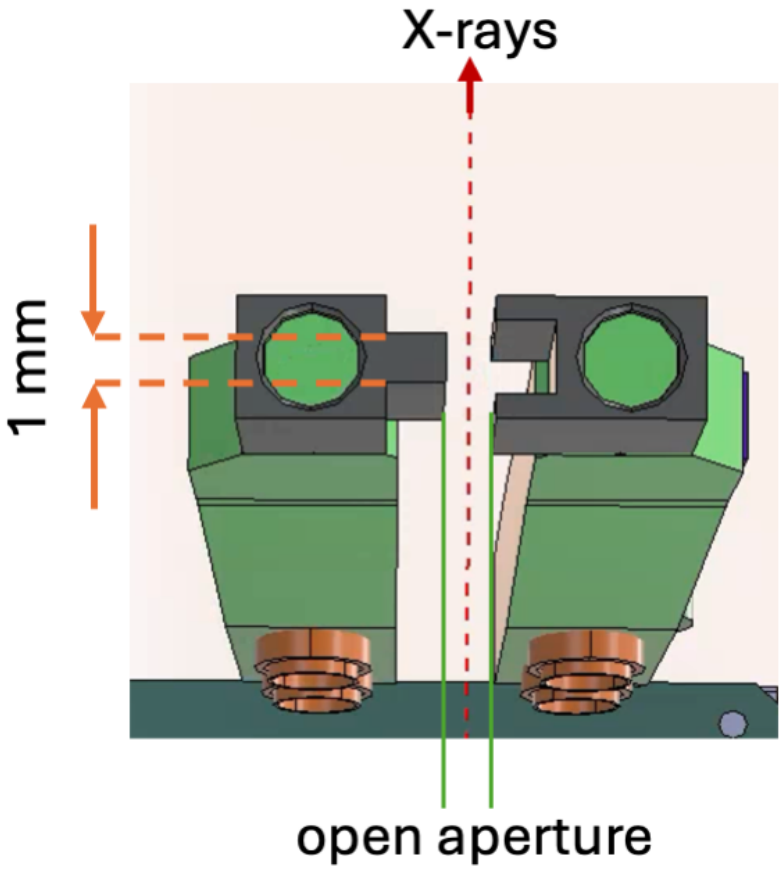

Figure 1: 3D rendering showing a close-up view of the shutter blade geometry. The X-rays propagade through the two blades, each of which provides a thickness of 1 mm tungsten material.

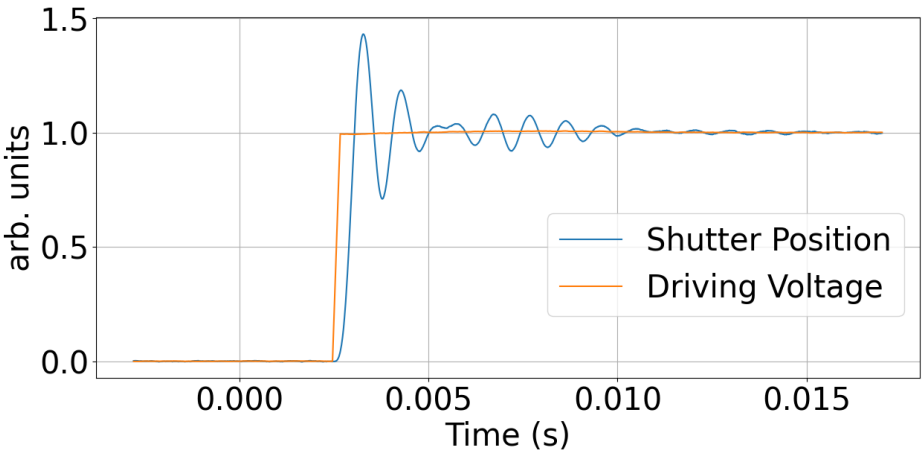

Figure 2: Behavior of the shutter when controlled with an edge driving voltage (orange). The position of the shutter blade (blue) shows clear oscillations due to mechanical excitation of resonance frequencies.

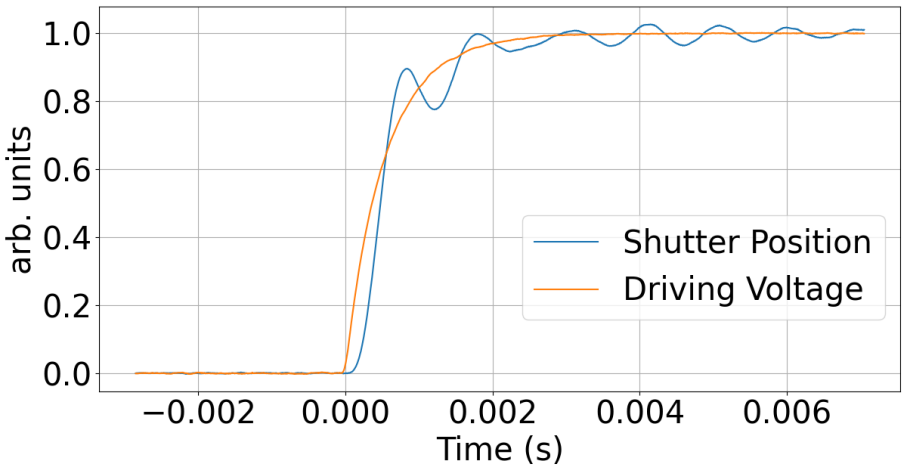

Figure 3: Behavior of the shutter when controlled with a low-pass filtered edge driving voltage (orange). The position of the shutter blade (blue) shows clear oscillations due to mechanical excitation of resonance frequencies.

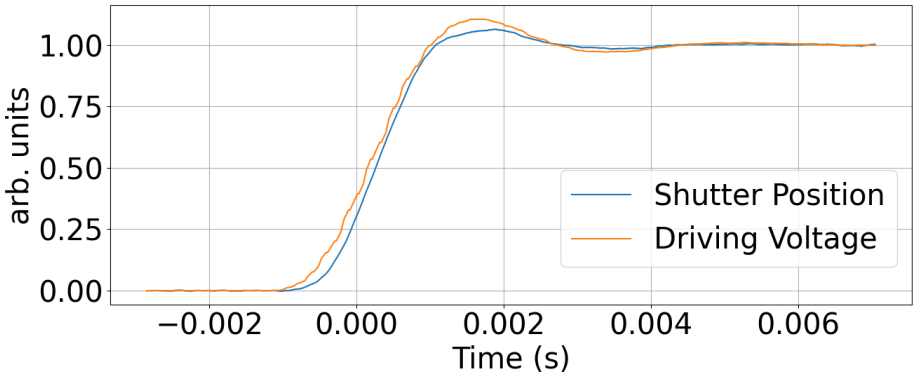

Figure 4: Behavior of the shutter when controlled with a synthesized driving voltage (orange). The position of the shutter blade (blue) smoothly follows the drive signal and no oscillations are excited.

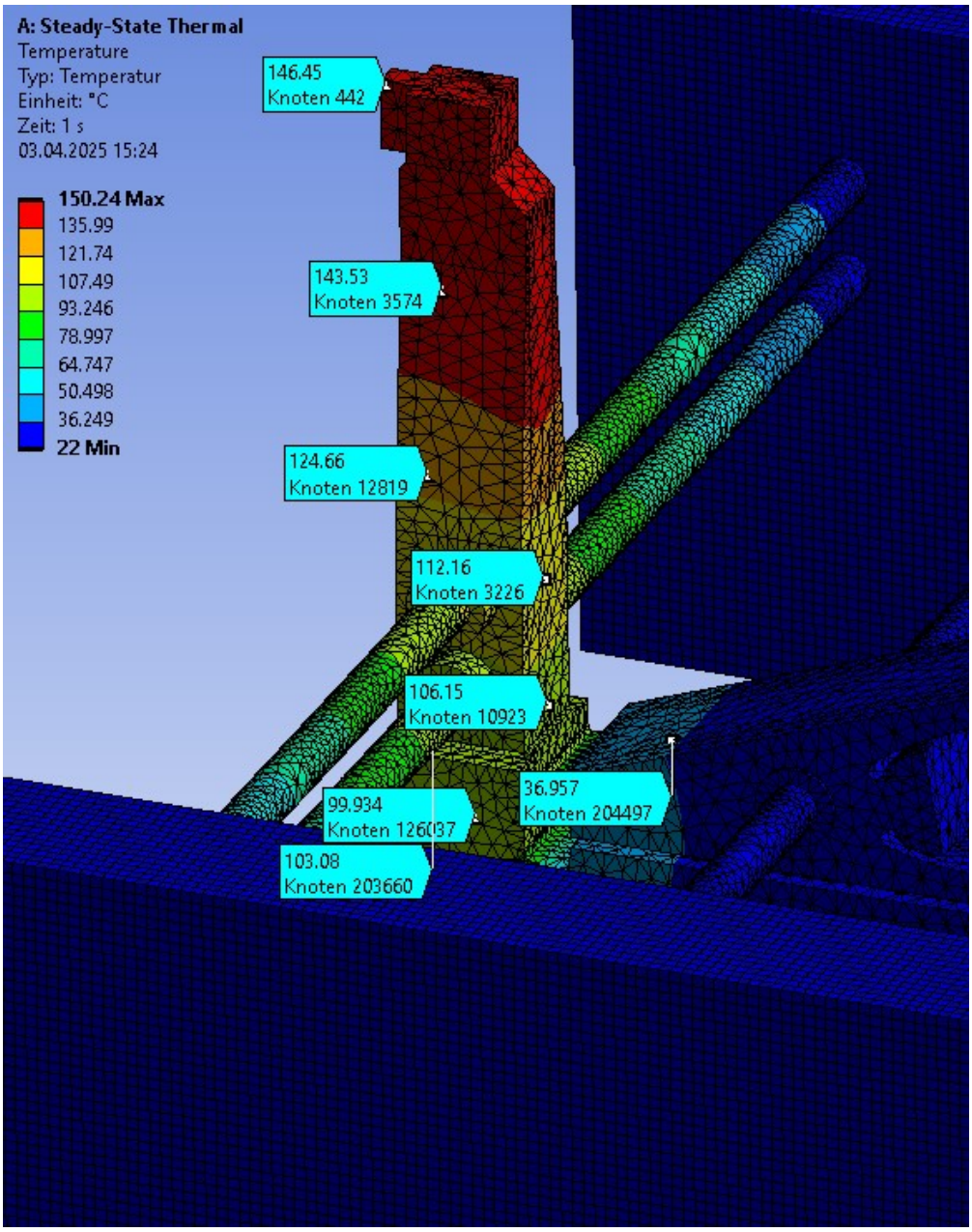

Figure 5: Thermal simulation of shutter temperatures with cooling water at 22 °C and a thermal load of 12 W incident power to a single shutter blade.

Python code to compute the drive signal

```
import numpy as np
import scipy.signal as signal
import matplotlib.pyplot as plt

```

```
# Parameters
sampling_rate = 10000 #Hz
notch_frequencies = [790]  # Hz
quality_factor = 50    # Qfactor Notch-Filter
lowpass_cutoff = 300  # Cutoff-Frequenz if the Low-Pass Filter

# Duration of the signal
duration = 1  # seconds
time = np.arange(0, duration, 1/sampling_rate)  # time vector

# Create the new input signal: 0 -> 1 -> 0 (sharp transitions)
hold_time = 0.5   # Time the signal stays at 1

# Create the signal: 0 to 1 transition, hold at 1, 1 to 0 transition
input_signal = np.zeros_like(time)
start_index = int(0.25 * sampling_rate)  # Start 250 ms into the signal
end_index = start_index + int(hold_time * sampling_rate)  # End after 500 ms
input_signal[start_index:end_index] = 0.7

# Zero out the initial transient before the step input
transient_duration = 0.1  # Duration to zero the initial part, e.g., 100 ms
transient_samples = int(transient_duration * sampling_rate)
input_signal[:transient_samples] = 0

# Design des Low-Pass Filters (Causal Filter with lfilter)
nyquist = 0.5 * sampling_rate
lowpass_cutoff_normalized = lowpass_cutoff / nyquist
b_lp, a_lp = signal.butter(4, lowpass_cutoff_normalized, btype='low')

# Apply Low-Pass Filters with lfilter (causal filter)
smoothed_signal = signal.lfilter(b_lp, a_lp, input_signal)

# Apply Notch-Filters (Causal Filter with lfilter)
filtered_signal = smoothed_signal.copy()

for notch_frequency in notch_frequencies:
    # Normalize Frequencies to nyquist
    low = (notch_frequency - 1) / nyquist
    high = (notch_frequency + 1) / nyquist
```

```
50
51      # Design Notch-Filters
52      b, a = signal.iirnotch(notch_frequency / nyquist, quality_factor)
53
54      # Apply
55      filtered_signal = signal.lfilter(b, a, filtered_signal)
56
57  # Frequency is set to sampling rate
58  freq_hz = 1
59  # Total points is the total number of samples in the signal
60  points = len(time)
61
62  header = [
63      "Data,Name,Freq(Hz),Ampl(VPP),Offset(VDC),Points",
64      f"0.0,,{freq_hz},0.7,0.0,{points}"
65  ]
66  # Export the input signal to a CSV file with metadata and proper formatting
67  filename = 'filtered_signal.csv'
68  with open(filename, 'w') as f:
69      # Write the header
70      for line in header:
71          f.write(line + '\n')
72
73      # Write the time and signal values (one value per line)
74      np.savetxt(f, np.column_stack((filtered_signal)), \
75             delimiter=',\n', fmt='%0.8f', \
76             header="", comments="")
77
78
79  header = [
80      "Data,Name,Freq(Hz),Ampl(VPP),Offset(VDC),Points",
81      f"0.0,,{freq_hz},0.7,0.0,{points}"
82  ]
83  # Export the input signal to a CSV file with metadata and proper formatting
84  filename = 'input_signal.csv'
85  with open(filename, 'w') as f:
86      # Write the header
87      for line in header:
88          f.write(line + '\n')
```

```
89
90      # Write the time and signal values (one value per line)
91      np.savetxt(f, np.column_stack((input_signal)), \
92             delimiter=',\n', fmt='%0.8f', \
93             header="", comments="")
94
95
96  # Visualize
97  plt.figure(figsize=(10, 8))
98
99  # Time-domain plots
100 plt.subplot(3, 1, 1)
101 plt.plot(time, input_signal, label="Input Signal (0 -> 1 -> 0)")
102 plt.title("Input Signal: 0 -> 1 -> 0 Transition with 500 ms at 1")
103 plt.xlabel("Time [s]")
104 plt.ylabel("Amplitude")
105 plt.grid(True)
106
107 plt.subplot(3, 1, 2)
108 plt.plot(time, filtered_signal, label="Filtered Signal", color='r')
109 plt.title("Signal After Low-Pass and Notch Filters (Causal)")
110 plt.xlabel("Time [s]")
111 plt.ylabel("Amplitude")
112 plt.grid(True)
113
114 # Frequency response of the notch filters (new subplot)
115 plt.subplot(3, 1, 3)
116
117 for notch_frequency in notch_frequencies:
118     # Design of the notch filter for visualization
119     b, a = signal.iirnotch(notch_frequency / nyquist, quality_factor)
120     w, h = signal.freqz(b, a, worN=2000)
121
122     # Plot the frequency response
123     plt.plot(w * nyquist / np.pi, abs(h), label=f'Notch Filter at {notch_frequency} Hz')
124
125 plt.title("Frequency Response of Notch Filters")
126 plt.xlabel("Frequency [Hz]")
127 plt.ylabel("Gain")
```

```
128 plt.grid(True)
129 plt.legend()
130 plt.tight_layout()
131 plt.show()
```
